# Mechanism of protective effect of xuan-bai-cheng-qi decoction on LPS-induced acute lung injury based on an integrated network pharmacology and RNA-sequencing approach

**DOI:** 10.1186/s12931-021-01781-1

**Published:** 2021-06-28

**Authors:** Huahe Zhu, Shun Wang, Cong Shan, Xiaoqian Li, Bo Tan, Qilong Chen, Yunxiang Yang, Hongji Yu, Aidong Yang

**Affiliations:** 1 School of Basic Medical Science, Shanghai University of Traditional Chinese Medicine, Shanghai, 201203 China; 2 Shuguang Hospital Affiliated to Shanghai University of Traditional Chinese Medicine, Shanghai, 201203 China; 3 Center for Research and Interdisciplinary, Shanghai University of Traditional Chinese Medicine, Shanghai, 201203 China; 4 College of Traditional Chinese Medicine, Tianjin University of Traditional Chinese Medicine, Tianjin, 301617 China

**Keywords:** Xuan-bai-cheng-qi decoction, Acute lung injury, Network pharmacology, Gene sequencing, Mechanism

## Abstract

Xuan-bai-cheng-qi decoction (XCD), a traditional Chinese medicine (TCM) prescription, has been widely used to treat a variety of respiratory diseases in China, especially to seriously infectious diseases such as acute lung injury (ALI). Due to the complexity of the chemical constituent, however, the underlying pharmacological mechanism of action of XCD is still unclear. To explore its protective mechanism on ALI, firstly, a network pharmacology experiment was conducted to construct a component-target network of XCD, which identified 46 active components and 280 predicted target genes. Then, RNA sequencing (RNA-seq) was used to screen differentially expressed genes (DEGs) between ALI model rats treated with and without XCD and 753 DEGs were found. By overlapping the target genes identified using network pharmacology and DEGs using RNA-seq, and subsequent protein–protein interaction (PPI) network analysis, 6 kernel targets such as vascular epidermal growth factor (VEGF), mammalian target of rapamycin (mTOR), AKT1, hypoxia-inducible factor-1α (HIF-1α), and phosphoinositide 3-kinase (PI3K) and gene of phosphate and tension homology deleted on chromsome ten (PTEN) were screened out to be closely relevant to ALI treatment. Verification experiments in the LPS-induced ALI model rats showed that XCD could alleviate lung tissue pathological injury through attenuating proinflammatory cytokines release such as tumor necrosis factor (TNF)-α, interleukin (IL)-6, and IL-1β. Meanwhile, both the mRNA and protein expression levels of PI3K, mTOR, HIF-1α, and VEGF in the lung tissues were down-regulated with XCD treatment. Therefore, the regulations of XCD on PI3K/mTOR/HIF-1α/VEGF signaling pathway was probably a crucial mechanism involved in the protective mechanism of XCD on ALI treatment.

**Supplementary Information:**

The online version contains supplementary material available at 10.1186/s12931-021-01781-1.

## Background

Acute lung injury (ALI) and its most severe form acute respiratory distress syndrome (ARDS) are the leading causes of acute respiratory failure in critically ill patients, with a mortality rate of 30–40% over the past 2 decades [1]. ALI/ARDS is increasingly recognized as a heterogeneous syndrome with alveolar epithelial and lung endothelial injuries caused by uncontrolled inflammation in the lung, pulmonary edema, and hyaline membrane formation [2]. Currently, the primary treatment is supportive therapy including mechanical ventilation and fluid management based on the underlying diseases and clinical care [3]. Effective pharmacological therapeutics that could delay the progress and long-term prognosis of ALI/ARDS are still lacking [1, 3]. Instead, an interest in alternative and natural therapies for ALI/ARDS treatment has been growing [4].

Xuan-bai-cheng-qi decoction (XCD) is a classic traditional Chinese medicine (TCM) prescription for the treatment of respiratory diseases in China [5, 6]. It contains four constituents, which are the mineral-based *Gypsum Fibrosum* and the herbs *Rheum officinale Baill*, *Semen Armeniacae Amarum*, and *Trichosanthes kirilowii Maxim* [7]. XCD could effectively improve disease symptoms and prognosis of ALI patients by protection of pulmonary function and alleviation of excessive inflammatory responses and tissue injuries with little adverse reactions [8, 9]. Moreover, XCD is recommended by Chinese health authorities as an alternative therapy for severe infectious pulmonary diseases caused by influenza and severe acute respiratory syndrome coronavirus 2 (SARS-CoV-2) [10, 11].

Several experimental studies have showed that XCD could treat ALI effectively through suppressing inflammatory responses and modulating the immune responses. For example, XCD could down-regulate the expression of cluster of differentiation 14 (CD14), LPS-binding protein (LBP), innate immune signal transduction adaptor MyD88, nuclear factor-kB (NF-kB), and Toll-like receptor 4 (TLR4) in lung tissues [12–14]. However, the mechanisms of protective effect of XCD in the treatment of ALI remains unclear.

TCM and other herbal medicines are characterized by multiple chemical components, multiple targets and multiple effects. Network pharmacology constructs the complex relationships between each components, targets, diseases, and molecular pathways [15, 16]. High-throughput RNA sequencing technology (RNA-seq) is a robust transcriptional screening technology, which could identify differentially expressed genes (DEGs) through comparisons of different conditions, such as normal and disease states [17, 18]. Thus, it is a beneficial strategy to combine the network pharmacology and RNA-seq technologies to explore the potential active ingredients and molecular mechanism of TCM and other herbal medicines [19].

Therefore, in the study we investigated the mechanism of protective effect of XCD on LPS-induced ALI model rats using an integrated network pharmacology and RNA-seq approach. Furthermore, the molecular mechanism of XCD was validated in ALI model rats.

## Methods

### Candidate compounds of XCD

The chemical compounds from the four medicines of XCD were intensively searched from both TCM Systems Pharmacology Database (TCMSP) [20] and Integrative Pharmacology-based Research Platform of TCM (TCMIP) [21]. Then, the candidate compounds were chosen among them with the favorable properties of absorption, distribution, metabolism, and excretion (ADME), in which the predicted oral bioavailability (OB) ≥ 30% and drug-likeness (DL) ≥ 0.18 were accepted as recommendation [22].

### Candidate targets related to XCD and ALI

The compound-related targets of XCD were collected from TCMSP with each candidate compound. The Human Gene Database (GeneCards) [23] and UniProt [24] were used for standardizing the names of target proteins to Homo sapiens. The targets failed to meet the condition were not selected for further analysis. Meanwhile, the disease-related targets were collected from three databases as follows: GenCards, Online Mendelian Inheritance in Man (OMIM) database [25], and DisGeNET [26]. Two keywords “acute lung injury” and “acute respiratory distress syndrome” were used for searching, and only “Homo sapiens” proteins linked to the disease were selected. Finally, the candidate targets were obtained from the overlaps between the compound-related targets and the disease-related targets.

### Compound-target network construction

The candidate compound-candidate target network (cC-cT network) was constructed by linking the candidate compounds to the candidate targets mentioned above using Cytoscape software (version 3.7.2). In the network, molecular species such as compounds and proteins were represented as nodes, and interactions between those molecular species were represented as edges.

### Pathway analyses

Functional annotation of target genes was analyzed using the online Database for Annotation, Visualization, and Integrated Discovery (DAVID, version 6.8) [27] and Kyoto Encyclopedia of Genes and Genomes (KEGG) pathway [28]. The significantly terms were defined as P < 0.05 and gene sets containing more than five genes. The Gene Ontology (GO) analysis, including biological processes (BP), cell composition (CC), and molecular functions (MF) was used to analyze the predicted targets.

### mRNA-sequencing

The lung tissue samples in three groups (n = 3 for each) for mRNA-sequencing (mRNA-seq) were collected as described below in the animal experiments section. These lung tissue sample were conducted using a CloudSeq mRNA enrichment kit and Illumina HiSeq sequencer (Thermo Fisher Scientific, Waltham, MA, USA) by Cloud-Seq Biotech Ltd. (Shanghai, China). Differentially expressed genes (DEGs) between the ALI model rats treated with and without XCD were identified using Cuffdiff software (part of the Cufflinks software). The thresholds of the DEGs were set as fold-change (FC) log |FC| ≥ 1.0 and P ≤ 0.05 with a fragments per kilobase million (FPKM) value ≥ 0.1, and at least one criterion was expected to be satisfied. Differentially expressed mRNA clustering was performed using FPKM values with the heat map function of R package.

### Protein–protein interaction (PPI) analyses and kernel target genes

The overlap targets between the candidate target genes and the DEGs were conducted by Venny2.0 [29] and identified as the key target genes, which were used to construct a PPI network by String database [30].The parameters of the PPI network, such as betweenness centrality (BC), closeness centrality (CC), degree (De), and topological coefficient (TC) were calculated. The kernel target genes were obtained from the top six key genes with the highest De values.

### Gene set enrichment analysis (GSEA)

To further confirm those screening key targets, we combined them to construct a self-defined geneset and conducted an enrichment analysis to determine whether these genes were biologically relevant to ALI by GSEA software (Version 4.1.0) [31]. The ALI related expression dataset (GSE5883) was obtained from GEO database (https://www.ncbi.nlm.nih.gov/geo/), in which 8 cultured human lung microvascular endothelial cells (HMVEC) samples treated with (ALI group, GSM114556–GSM114559, n = 4) or without LPS (Control group, GSM114568–GSM114571, n = 4) for 8 h were used for comparison. The normalized enrichment score (|NES|) ≥ 1.5, nominal p-value < 0.05 and a false discovery rate (FDR) q-value < 0.25 were considered significantly enriched.

### Animal experiments

Thirty male Wistar rats (weight 200 ± 20 g) were purchased from Shanghai Sippr-BK Laboratory Animals Co., Ltd. (Shanghai, China) and were maintained in an animal facility at 16–26 °C, with a light/dark cycle of 12/12 h and humidity of 40–70%. The rats were fed standard chow and provided with clean water ad libitum. Those rats were randomly divided into five groups (n = 6 in each group): normal, model (Lipopolysaccharide, LPS) group, XCD low- and high-dose, and dexamethasone (DEX) groups. The rats in the XCD low- and high-dose groups were intragastrically administered of XCD at 5 and 20 g crude herb/kg, respectively. The rats in the DEX group were administered 0.27 mg/kg DEX (based on the equivalent human dose [9]). Each drug was administered once daily for successive 5 days. Rats in both normal and model groups received the same volume of distilled water. Two hours after last dosing on day 5, all rats were injected with LPS (8 mg/kg) via the tail vein except for the normal group. Seven hours later, the rats were euthanized and collected bronchoalveolar lavage fluid (BALF) as previously reported [32]. All animal experiments were performed in accordance with the National Institutes of Health Guidelines for the Care and Use of Laboratory Animals and were approved by the Research Ethics Committee for Experimental Animal Center of Shanghai University of TCM (No.: PZSHUTCM19012502).

Lyophilized XCD powder was prepared by mixing *Gypsum fibrosum* (30 g), *R. officinale Baill* (6 g), *Semen Armeniacae Amarum* (12 g), and *T. kirilowii Maxim* (10 g) as previously reported [33]. The powder yield was 8.7%. For dosing, the XCD decoction was prepared as an aqueous solution containing 1 g crude herb mixture per 1 ml.

### Histological analysis

The upper lobe of the right lung was harvested, fixed in 4% paraformaldehyde, and embedded in paraffin wax. The embedded tissue was sectioned, stained with hematoxylin and eosin (HE) following a standard protocol, and then analyzed pathological changes under a light microscopy (AXIO Scope A1, ZEISS, Germany) [34].

### ELISA

TNF-α, IL-6 and IL-1β, in BALF were measured by ELISA following the manufacturer’s instructions (R&D Systems, Minneapolis, MN, USA).

### qRT-PCR

The mRNA expression of phosphoinositide 3-kinase (PI3K), phosphatase and tensin homolog (PTEN), AKT, mammalian target of rapamycin (mTOR), vascular epidermal growth factor (VEGF), hypoxia-inducible factor (HIF)-1α, and glyceraldehyde-3-phosphate dehydrogenase (GAPDH) in the lung tissues were detected by qRT-PCR. Total RNA was extracted from lung tissues with TRIzol according to the manufacturer’s instructions (EZBioscience, US), and the isolated RNA was reverse-transcribed into cDNA with a RevertAid First Strand cDNA Synthesis Kit (TAKARA, China). Quantitative real-time (qRT)-PCR was performed with the LightCycler 96 Real-Time PCR System (Roche) and RevertAid First Strand cDNA Synthesis Kit (TAKARA, China). The genes primer sequences (Additional file 1: Table S1) for qRT-PCR were designed by Shanghai Sangon Biotech Biological Co., Ltd (Shanghai, China) [35]. The qRT-PCR analysis for each sample was conducted in triplicate with 2^–ΔΔ^CT method. Expression levels were normalized against the reference gene GAPDH.

### Western blot analysis

The protein expression levels of p-PI3K, p-mTOR, HIF-1α, and VEGF in lung tissues were measured by western blot analysis. In brief, lung tissue samples were harvested and homogenized to extract the protein, which was quantified as described previously [36]. The same amount of total protein was separated using electrophoresis and voltage transmembrane potential. Then, the gel was incubated with primary antibodies against p-PI3K (ab182651, Abcam, 1:2000), p-mTOR (ab109268, Abcam, 1:1000), HIF-1α (ab179483, Abcam, 1:1000), or VEGF (ab32152, Abcam, 1:1000), followed by the secondary antibody each for an appropriate time. The gel imaging system (Bio-Rad Inc, USA) with Alpha View SA software (Protein Simple, CA, USA) was used for analysis. The ratio of the grayscale of the target strip to that of the internal reference strip was calculated as the relative expression level of the target protein.

### Immunohistochemical assay (IHC)

The protein expression levels of PI3K, AKT, PTEN, mTOR, VEGF, and HIF-1α, in lung tissues were measured by IHC. In brief, lung tissue samples were fixed in formalin, embedded in paraffin, and the 4 μm-thick sections were placed on positive-charged slides. After incubation with primary antibodies PI3K (ab140307, Abcam, 1:150), AKT (ab88050, Abcam, 1:100), PTEN (ab267787, Abcam, 1:2000), mTOR (ab134903, Abcam, 1:400), VEGF (ab32152, Abcam, 1:100), and HIF-1α (ab51608, Abcam, 1:100) for 30 min, the immune reaction was detected using a peroxidase-labeled secondary reagent, EnVision™ (K4011 for rabbit antibodies and K4007 for mouse antibodies, Dako). The immunostaining intensity of each protein was evaluated using the image mapping spectrometer (IMS) cell image analysis system (Shenteng Information Technology Co., Ltd, Shanghai, China). For each section, three non-overlapping fields of view were randomly selected under a high-power lens to calculate the positive area ratio (positive cell area/total area).

### Statistics

For bioinformatics analyses, the data were analyzed using the hypergeometric distribution test and Fisher’s exact test. The Benjamini–Hochberg method was used to correct the false discovery rate (FDR). Otherwise, the data were expressed as means ± standard deviation (SD) and analyzed using the statistical package for the social sciences (SPSS) 21.0 software (IBM, NY, USA). The means of multiple groups were compared with one-way analysis of variance (ANOVA), which is preliminary performed by checking homogeneity of variance, and then followed by the least-significant difference (LSD) method. A *P* < 0.05 was considered statistically significant.

## Results

### Active ingredients and target screening

A total of 206 compounds in XCD were selected from TCMIP and TCMSP databases. Screening on the two criteria that OB thresholds ≥ 0.3 and DL ≥ 0.18, identified 46 candidate compounds in XCD. 16 in *R. officinale Baill*, 19 *Semen Armeniacae Amarum* and 11 in *T. kirilowii Maxim* (Additional file 1: Table S2). Excluding three compounds which had no reported targets in the two databases or literature, 43 compounds mainly flavonoids and organic acids were considered as potential active ingredients (Table 1). Screening in the TCMIP and TCMSP database, 281 compound-related targets were obtained (Additional file 1: Tables S3). Meanwhile, selecting from the three databases, GenCards, OMIM, and DisGeNET, 6783 disease-related targets were obtained (Additional file 1: Table S4). The overlapping targets between the above two data sets (281 targets, Table S5) were identified as candidate targets for XCD treatments in ALI.

**Table 1:** The candidate compounds of XCD

No	Herbal medicine	Chemical	No	Herbal medicine	Chemical
1	*R. officinale Baill*	Eupatin	24	*Semen Armeniacae Amarum*	Stigmasterol
2	*R. officinale Baill*	Emodin	25	*Semen Armeniacae Amarum*	Glabridin
3	*R. officinale Baill*	Physciondiglucoside	26	*Semen Armeniacae Amarum*	Estrone
4	*R. officinale Baill*	Procyanidin B-5,3′-O-gallate	27	*Semen Armeniacae Amarum*	(+)-catechin
5	*R. officinale Baill*	Rhein	28	*Semen Armeniacae Amarum*	Mairin
6	*R. officinale Baill*	Sennoside E_qt	29	*Semen Armeniacae Amarum*	Liquiritin
7	*R. officinale Baill*	Torachrysone-8-O-beta-D-(6′-oxayl)-glucoside	30	*Semen Armeniacae Amarum*	Ziziphin_qt
8	*R. officinale Baill*	Toralactone	31	*Semen Armeniacae Amarum*	Licochalcone B
9	*R. officinale Baill*	Emodin-1-O-beta-D-glucopyranoside	32	*Semen Armeniacae Amarum*	Phaseol
10	*R. officinale Baill*	Sennoside D_qt	33	*Semen Armeniacae Amarum*	Machiline
11	*R. officinale Baill*	Daucosterol_qt	34	*Semen Armeniacae Amarum*	l-SPD
12	*R. officinale Baill*	Palmidin A	35	*Semen Armeniacae Amarum*	Glycyrol
13	*R. officinale Baill*	Beta-sitosterol	36	*T. kirilowii Maxim*	Mandenol
14	*R. officinale Baill*	Aloe-emodin	37	*T. kirilowii Maxim*	Linolenic acid ethyl ester
15	*R. officinale Baill*	Gallic acid-3-O-(6′-O-galloyl)-glucoside	38	*T. kirilowii Maxim*	Hydroxygenkwanin
16	*R. officinale Baill*	(−)-catechin	39	*T. kirilowii Maxim*	Diosmetin
17	*Semen Armeniacae Amarum*	Gondoic acid	40	*T. kirilowii Maxim*	karounidiol 3-o-benzoate
18	*Semen Armeniacae Amarum*	Diisooctyl succinate	41	*T. kirilowii Maxim*	Vitamin-e
19	*Semen Armeniacae Amarum*	(6Z,10E,14E,18E)-2,6,10,15,19,23-hexamethyltetracosa-2,6,10,14,18,22-hexaene	42	*T. kirilowii Maxim*	10α-cucurbita-5,24-diene-3β-ol
20	*Semen Armeniacae Amarum*	Sitosterol	43	*T. kirilowii Maxim*	Schottenol
21	*Semen Armeniacae Amarum*	CLR	44	*T. kirilowii Maxim*	7-oxo-dihydrokaro-unidiol
22	*Semen Armeniacae Amarum*	11,14-eicosadienoic acid	45	*T. kirilowii Maxim*	Spinasterol
23	*Semen Armeniacae Amarum*	Spinasterol	46	*T. kirilowii Maxim*	5-dehydrokarounidiol

### Candidate compound-candidate target network construction and target enrichment analysis

**Fig. 1:**
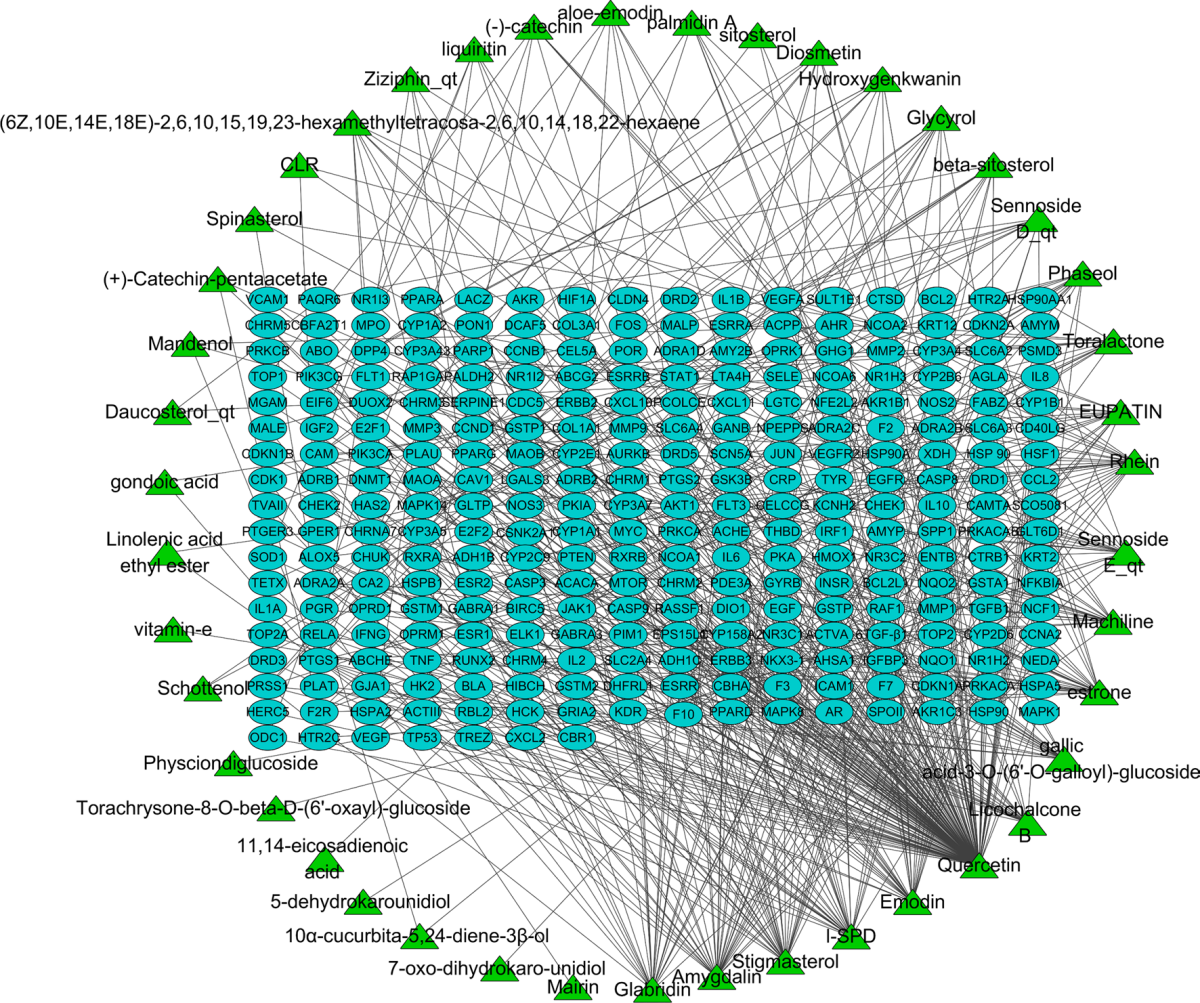
Network construction using 43 candidate compounds and 281 candidate target genes of xuan-bai-cheng-qi decoction (XCD). Green triangles and blue ovals represent candidate compounds and candidate target genes of XCD, respectively

The candidate compound-candidate target network was constructed by 43 candidate compounds and 281 candidate targets (Fig. 1, Additional file 1: Table S5). Quercetin, amygdalin, stigmasterol, L-SPD, and emodin were the five most relevant candidate compounds, while prostaglandin-endoperoxide synthase 2 (PTGS2), nuclear receptor coactivator 2 (NCOA2), prostaglandin-endoperoxide synthase 1 (PTGS1), progesterone receptor (PGR), androgen receptor (AR), calmodulin (CAM), factor X (F10), heat shock protein 90 (HSP90), nuclear receptor subfamily3, group C, member2 (NR3C2), and nitric oxide synthase 2 (NOS2) were the ten most relevant candidate targets.

**Fig. 2:**
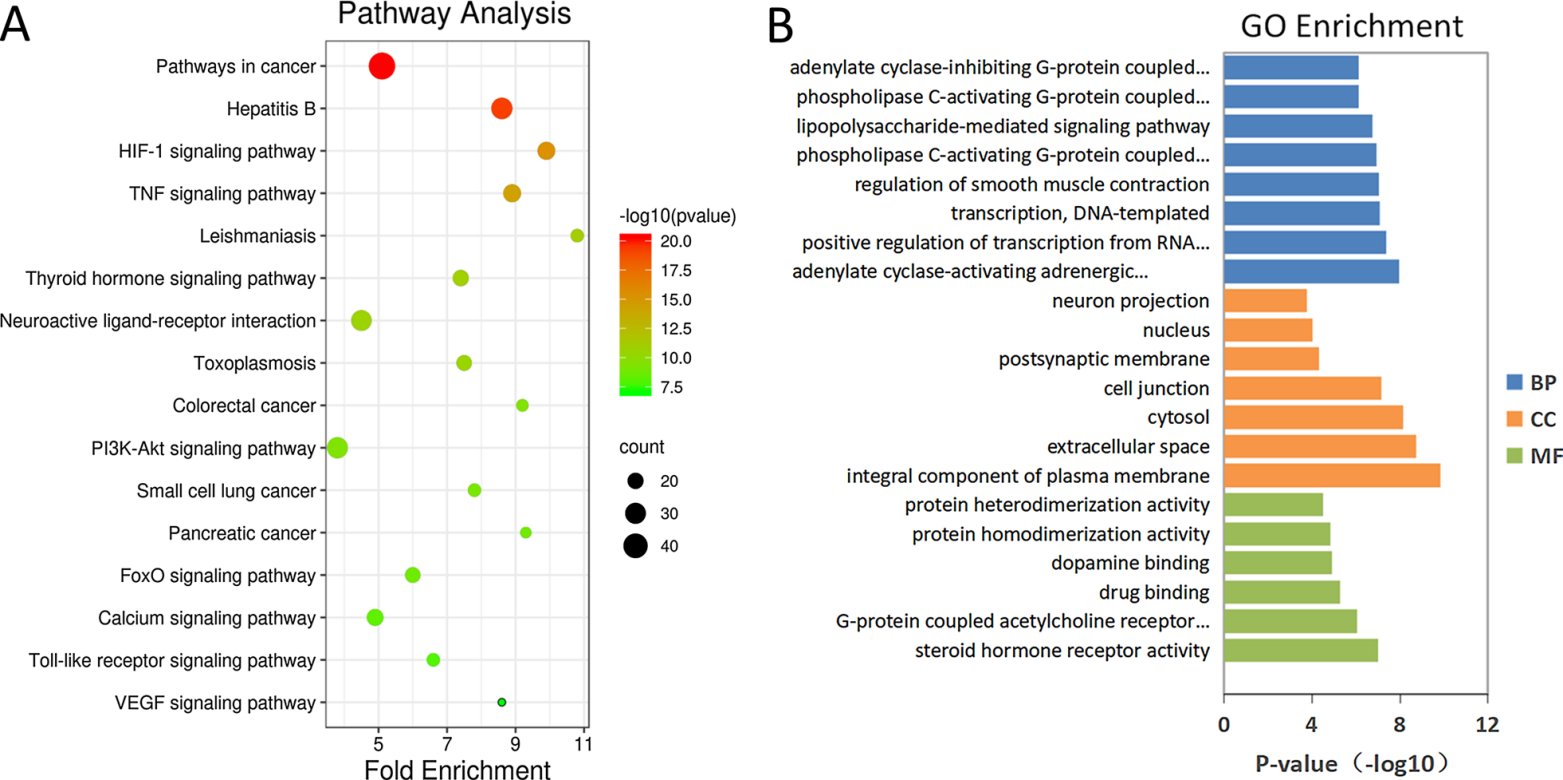
List of Gene Ontology (GO) and Kyoto Encyclopedia of Genes and Genomes (KEGG) pathway enrichment results in relation to the candidate target genes of xuan-bai-cheng-qi decoction (XCD)-treated acute lung injury (ALI). **A** The top 16 pathways were identified based on P values < 0.05. **B** The top 21 GO terms were identified based on P values < 0.01

Then, the functional enrichment analysis of 281 candidate targets was conducted using the DAVID program. The KEGG pathway analysis (Fig. 2A) showed that the target genes were mainly associated with pathways related with immunity and inflammation, such as cancer (prostate, bladder, colorectal, small cell lung, and pancreatic), hepatitis B and apoptosis, and a variety of signaling-related pathways, such as HIF-1α, tumor necrosis factor (TNF), VEGF, mTOR, Forkhead box O (FOXO), PI3K-AKT, nuclear factor (NF)-κB, and nucleotide-binding oligomerization domain (NOD)-like receptor. The GO analyses obtained enriched results of those candidate target genes (Fig. 2B), which showed that LPS-mediated signaling pathway, cellular response to hypoxia, positive regulation of angiogenesis, extrinsic apoptotic signaling pathway in the absence of a ligand and cell–cell signaling, activation of cysteine-type endopeptidase activity involved in apoptotic processes were closely related to the activities of XCD in ALI.

### DEGs identified by mRNA-seq and their pathway enrichment analysis

**Fig. 3:**
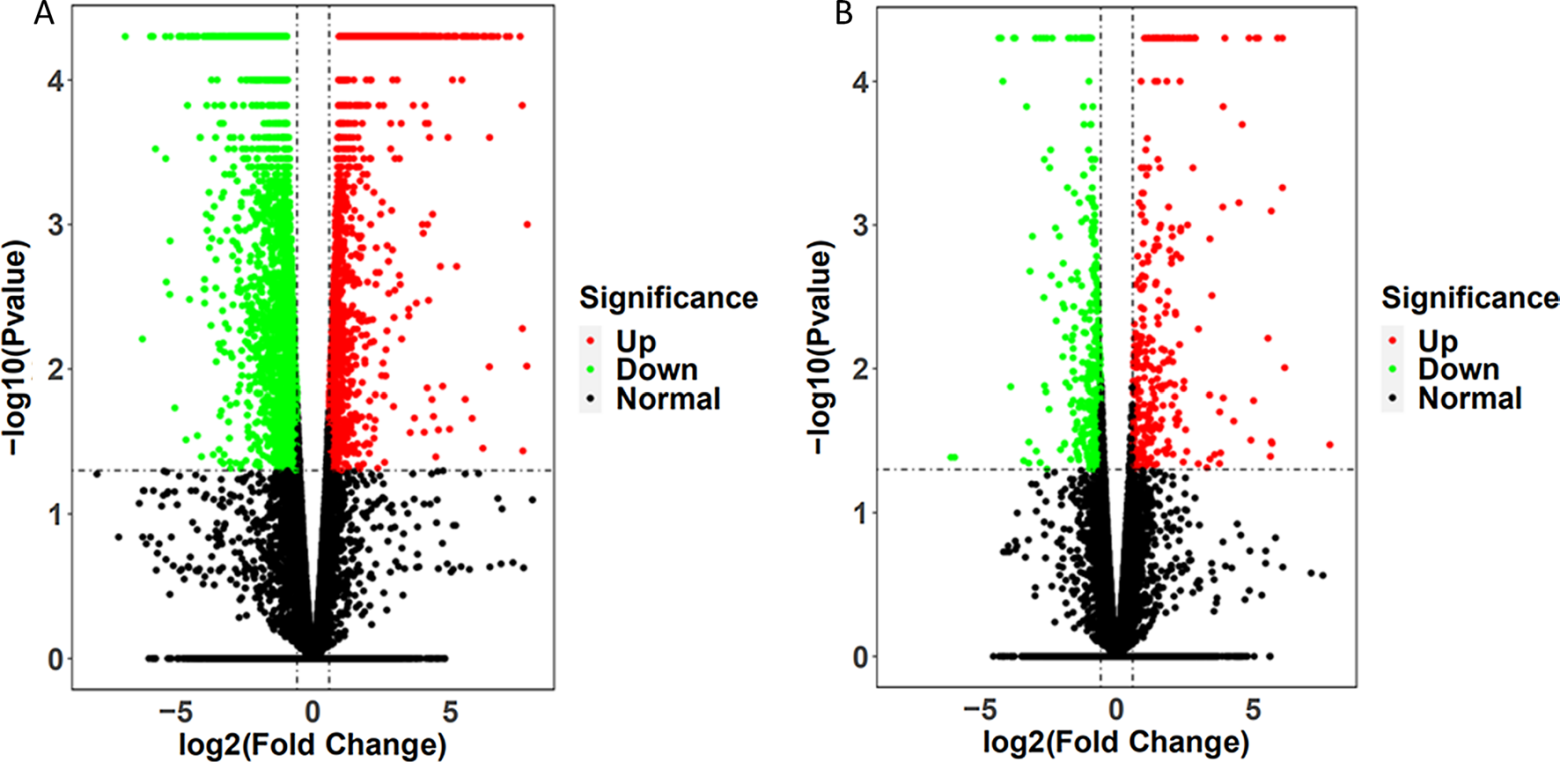
The differentially expressed genes between Model: Normal (**A**), XCD: Model (**B**) in lung tissue of LPS-induced ALI rat, the black dots represented genes without different expression between two group, the red dots showed that genes level was upregulated and the green dots was down-regulated

**Fig. 4:**
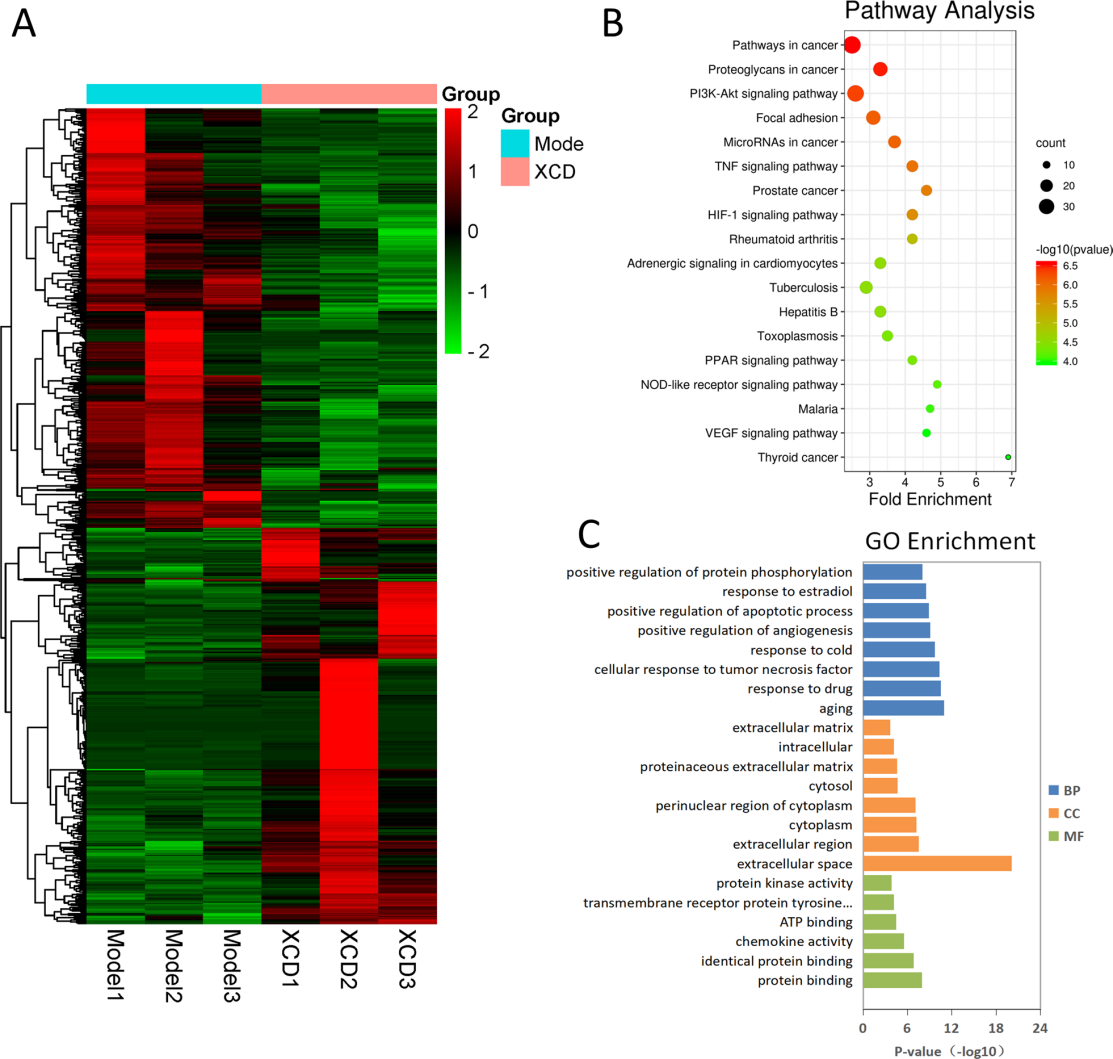
Differentially expressed gene (DEG) analysis of LPS induced acute lung injury (ALI) rat treated with or without xuan-bai-cheng-qi decoction (XCD). **A** Heatmap was created using DEGs. Red and green colors represent upregulated and downregulated expression of genes, respectively. Sixty-five gene targets obtained by cross-referencing key targets using high-throughput gene sequencing showed significant differences between model (rats treated with LPS only) and XCD groups. Distribution of biological functions of DEGs were **B** Kyoto Encyclopedia of Genes and Genomes (KEGG) pathways analyses, **C** biological process (BP) terms, molecular function (MF) terms, and cellular component (CC) terms in XCD-treated ALI rat model

The mRNA profiles of lung tissues of rats in both the model and XCD high-dose groups (n = 3) were determined using mRNA-seq. In all, 22,062 genes were identified, which were expressed in at least one sample (FPKM cut-off value 0.01). The number of expressed genes were 16,436 in the Normal group, 16,257 in the Model group, 16,423 in the XCD group. To determine the differentially expressed genes (DEGs), a P value < 0.05 which was detected by pairwise comparisons between the Model group and the Normal, XCD and Model groups was used as the screening criteria for gene expression in the Normal, Model, and XCD groups. Overall, 1085 upregulated and 1768 downregulated DEGs were identified in the Model vs Normal groups (Fig. 3A), and 485 upregulated and 448 downregulated DEGs were identified in the XCD vs. Model groups (Fig. 3B). Based on the criteria of log|FC| > 1 and P < 0.05, a total of 753 genes including 361 upregulated and 392 downregulated genes were identified between the Model group and XCD group (Additional file 1: Table S6). The heat map function of the R package used for analyzing the DEGs by using the FPKM value, which showed a significant difference in gene expression between the Model group and XCD group (Fig. 4A, Additional file 1: Table S7).

The interactions between the DEGs were analyzed using functional enrichment with the DAVID program. KEGG pathway and GO enrichment analysis showed that the DEGs were mainly associated with Pathways in cancer, Proteoglycans in cancer, Focal adhesion, MicroRNAs in cancer, Prostate cancer, Rheumatoid arthritis, Adrenergic signaling in cardiomyocytes, Tuberculosis, Hepatitis B, Toxoplasmosis, Malaria, Thyroid cancer, and a variety of signal-related pathways, such as PI3K-AKT, TNF, HIF-1, PPAR, NOD-like receptor, VEGF signaling pathways (Fig. 4B). Interestingly, some KEGG pathways of DEGs were overlapped with candidate target genes, which indicated some key target genes might exist, and the GO results of the functional analysis were also generally consistent with XCD network pharmacology (Fig. 4C). Taken together, the DEGs were closely related to the pulmonary inflammation storm and pulmonary edema of LPS-induced ALI, such as positive regulation of angiogenesis, positive regulation of apoptotic process, positive regulation of protein phosphorylation, negative regulation of cell proliferation, response to LPS, inflammatory responses and response to hypoxia.

### PPI network construction and identification of kernel targets

**Fig. 5:**
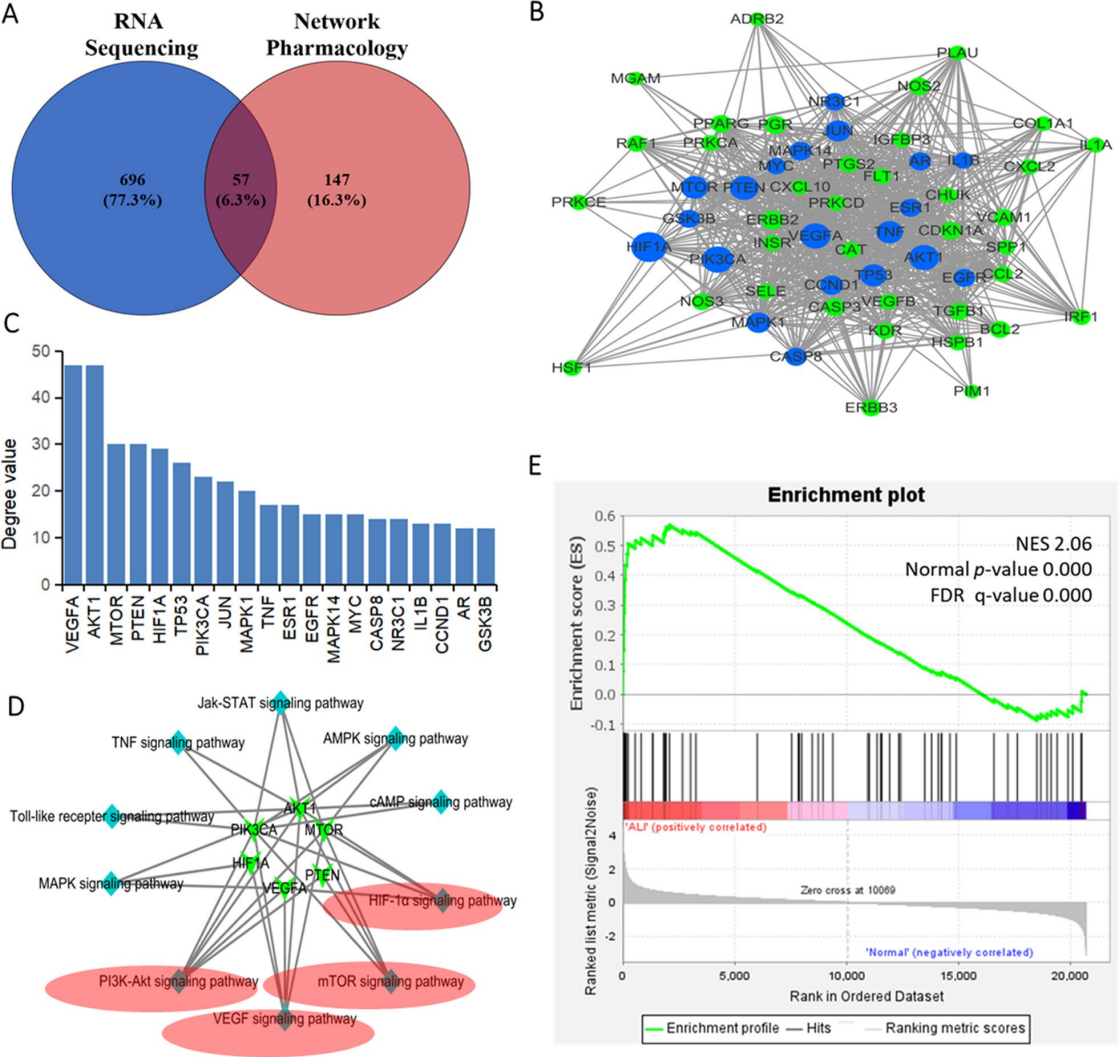
Screening and analysis of key targets and kernal targets. **A** Venn diagram between target genes from network pharmacology and differentially expressed genes (DEG) from RNA-sequencing. **B** Protein–protein interaction (PPI) network constructed using key target genes, the blue dots represent the top 20 targets of degree value in the network, and the green dots represent the rest of the targets with a lower degree value. **C** The top 20 target genes with degree values above the average value. **D** Kyoto Encyclopedia of Genes and Genomes (KEGG) pathway-target network constructed using kernel targets and related signaling pathways. Red ellipses indicate overlapping signaling pathways between target-pathway network and kernel target pathway, which include hypoxia-inducible factor (HIF)-1α, phosphoinositide 3-kinase (PI3K)-AKT, mammalian target of rapamycin (mTOR), and vascular epidermal growth factor (VEGF) signaling pathways. **E** The self-defined geneset containing 57 target genes was significantly enriched in the expression dataset for human lung microvascular endothelial cells (HMVEC) stimulated with or without LPS for 8 h

Overlapping the candidate targets from network pharmacology analyses and the DEGs from RNA-seq, 57 genes were found and considered as key targets (Fig. 5A, Additional file 1: Table S8). Then, the shared 57 targets were used to construct the PPI network (Fig. 5B), which contained 57 nodes (of which there are no isolated targets) and 333 edges with an average nodal degree value of 11.6. There were 20 targets with degree values greater than the average value, which might be the key targets of XCD in ALI treatment (Fig. 5C). The top six genes with the highest nodal degree values, namely PIK3CA, MTOR, AKT1, PTEN, HIF1A, and VEGFA, were selected as kernel targets (Table 2). To further screen the corresponding pathways of the kernel targets, the kernel target-pathway network was constructed and KEGG analyses were conducted, indicating that the PI3K-AKT, mTOR, HIF-1α, and VEGF signaling pathways were closely related to these kernel genes (Fig. 5D, Additional file 1: Table S9).

**Table 2:** Kernel targets screened from protein–protein interaction (PPI) network

Gene	Closeness centrality	Degree	Betweenness centrality	Topological coefficient
*AKT1*	0.8615	47	0.2180	0.2200
*VEGFA*	0.8485	47	0.2348	0.2197
*PTEN*	0.6747	30	0.0429	0.2842
*MTOR*	0.6667	30	0.0603	0.2722
*HIF1A*	0.6588	29	0.0436	0.2861
*PIK3CA*	0.6222	23	0.0282	0.2988

### The expression of key target genes in HMVEC stimulated with or without LPS

The geneset containing 57 target genes was significantly (NES = 2.02, normal p-value = 0.000 and FDR q-value = 0.000) enriched in the expression dataset established by HMVEC samples stimulated with LPS for 8 h (Fig. 5E and Additional file 1: Table S10) through GSEA analysis, which implied that the 57 target genes involved in pathogenesis of ALI. Moreover, the changes of these genes in the leading edge subset were correlated with those in rat lung tissues by RNA-seq analysis.

### XCD treatment alleviated the pathological changes and inflammatory cytokines levels induced by LPS

**Fig. 6:**
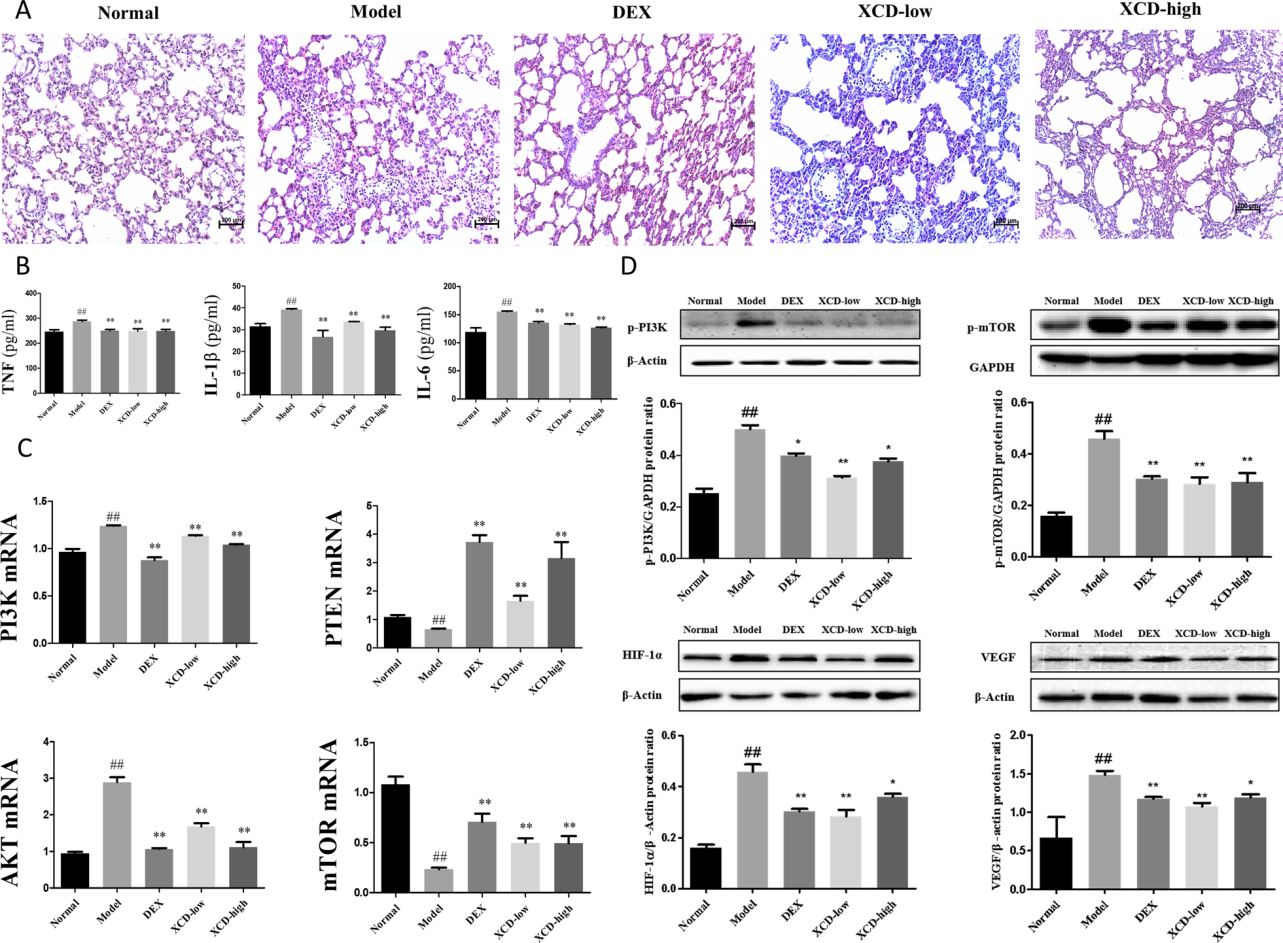
Animal experiment validation. **A** Histopathological examination was used to verify established acute lung injury (ALI) model and observed treatment effects. Normal, Model, dexamethasone (DEX), and low- and high-dose xuan-bai-cheng-qi decoction (XCD) groups. **B** TNF-α, IL-6 and IL-1β in BALF were analyzed by ELISA. Each bar represents the mean ± SD. ^##^P < 0.01 compared with Normal. **P < 0.01 compared with Model. **C** The results of qRT-PCR were performed to analyze PTEN, PI3K, AKT and MTOR mRNA expression in the lung tissues. **D** The expressions of phosphorylated-phosphoinositide 3-kinase (p-PI3K), phosphorylated-mammalian target of rapamycin (p-mTOR), hypoxia-inducible factor (HIF)-1α, and vascular epidermal growth factor (VEGF) in lung tissues by western blots (means ± standard deviation, n = 6). ^##^P < 0.01, compared to control group; **P < 0.01 and *P < 0.05 compared to model group

XCD significantly alleviated lung tissue injury as well as inflammatory cell infiltration induced by LPS, which were similar as treated with dexamethasone (DEX) (Fig. 6A). In the Model group, lung tissue showed significant interstitial pneumonia and edema around the small interstitial vessels, whereas in both XCD treatment pathological changes of lung tissue such as alveolar dilatation, emphysema, and interstitial pneumonia were found obviously decrease as well as those in DEX group. Also, XCD significantly decreased the levels of proinflammatory cytokines such as TNF-α, IL-6 and IL-1β in BALF (P < 0.05, respectively) (Fig. 6B).

### XCD treated ALI by inhibiting the PI3K/AKT/mTOR signaling pathways

**Fig. 7:**
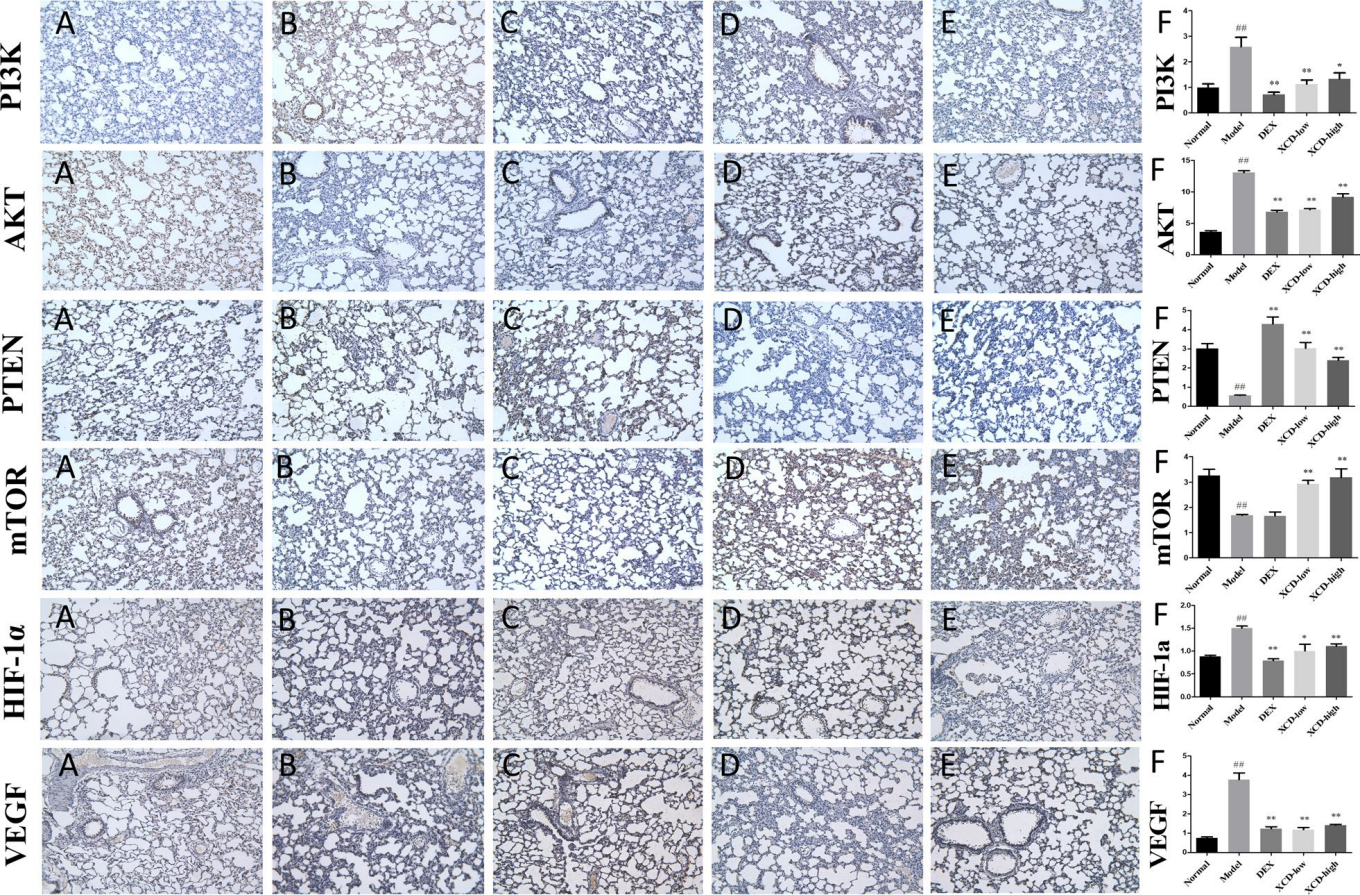
Immunohistochemical analysis of xuan-bai-cheng-qi decoction (XCD)-treated lipopolysaccharide (LPS)-induced acute lung injury (ALI) in rats. Expression levels of phosphatase and tensin homolog (PTEN) and mammalian target of rapamycin (mTOR) were upregulated, while those of phosphoinositide 3-kinase (PI3K), vascular epidermal growth factor (VEGF), and hypoxia-inducible factor (HIF)-1α were downregulated. **A** Normal, **B** model, **C** dexamethasone (DEX), and **D** low- and **E** high-dose XCD groups. **F** Protein expression level (%). ^##^P < 0.01, compared to control group and **P < 0.01 and *P < 0.05, compared to model group

The mRNA and protein expressions of the six kernel targets, such as PI3K, AKT, PTEN, mTOR, VEGF, and HIF1A, in lung tissues were investigated. By qRT-PCR analyses, the mRNA expressions of PTEN and mTOR were decreased (P < 0.05, respectively), and the mRNA expression of PI3K and AKT were increased (P < 0.05, respectively) with LPS stimulation. In contrast, XCD treatments with either low-dose or high-dose could significantly recover the mRNA expressions of the four targets (P < 0.05, respectively) (Fig. 6C). WB analyses showed similar changes in the protein expressions of kernel targets proteins in those groups (Fig. 6D). Meanwhile, the protein expressions of phosphorylated-mTOR (p-mTOR), phosphorylated-PI3K (p-PI3K), HIF-1α, and VEGF in lung tissues were significantly increased with LPS stimulation (P < 0.05, respectively). In contrast, the expressions of those proteins with two doses of XCD treatments showed significantly decrements, respectively (P < 0.05, respectively) (Fig. 6D). The observations by IHC analyses showed similar trends as those in WB analyses. The expressions of PI3K, AKT, HIF-1α, and VEGF in lung tissues were significantly increased (P < 0.01, respectively), while the expressions of PTEN and mTOR were significantly decreased (P < 0.01, respectively) with LPS stimulation. In contrast, the expressions of PI3K, AKT, PTEN, mTOR, HIF-1α, and VEGF, could be recovered significantly with both two doses of XCD treatments (P < 0.05 or P < 0.01, respectively), as well as those of DEX treatment except for mTOR (Fig. 7).

## Discussion

As a Chinese classical prescription, XCD is wildly used to treat a variety of respiratory diseases in China for a long history, especially to seriously infectious diseases such as ALI. Several studies have revealed that XCD and its component herbs could effectively suppress inflammation responses and modulating the immune processes in ALI [8, 37]. However, due to the complexity of the chemical constituents of XCD in which at least hundreds of compounds were found, the mechanisms of protective effect of XCD in the treatment of ALI remains unclear [38]. Recent studies have shown that RNA sequencing and network pharmacology can effectively enrich for key targets in a wide range of genes relevant to the treatment of diseases associated with herbal compounds [39]. In our study, we investigated the protective mechanism of XCD for ALI treatment using a combined strategy comprised of network pharmacology and RNA-seq technologies. With the help of network pharmacological analysis, 46 main active ingredients and 281 ALI-related targets were obtained. Meanwhile, RNA-seq analysis of lung tissues between ALI rats treated with and without XCD yielded other 753 DEGs. Overlapping the two target sets found in both technologies, 57 key target genes were obtained, and confirmed by a GSEA analysis with expression datasets for HMVEC samples stimulated with or without LPS for 8 h from GEO database. Then, through PPI construction and analysis, 6 kernel targets, namely AKT, PI3KCA, MTOR, HIF1A, VEGFA, and PTEN, were screened out to be closely relevant to XCD treatment on ALI. Following KEGG pathway analysis revealed that four important pathways were involved, namely PI3K, AKT, mTOR, HIF-1α, TNF and VEGF signaling pathways.

The animal experiments confirmed that XCD treatment could alleviate lung tissue pathological injury by LPS stimulation through attenuating the proinflammatory cytokines release (including TNF-α, IL-6 and IL-1β). Moreover, the pulmonary protein expression of AKT, PI3K, p-PI3K, mTOR, p-mTOR, HIF-1α, VEGF in LPS induced ALI rats were significantly downregulated with XCD treatment, while the expression of PTEN were significantly upregulated by both WB and IHC analyses. The mRNA levels for the corresponding genes (PI3K, AKT, mTOR, PTEN) also exhibited similar trends as that of protein expressions. Therefore, PI3K/ mTOR / HIF-1α / VEGF signaling pathway was probably a key mechanism involving in anti-inflammatory effects of XCD on ALI treatment.

PI3K/AKT signaling pathway plays an important role in alleviating lung inflammation by reducing neutrophil apoptosis [40]. It could regulate mTOR, an important serine threonine protease, to decreases the pulmonary inflammatory injuries such as barrier destruction, pulmonary edema, and airway inflammation through regulating cell survival under oxidative stress and preventing against inflammation responses [41, 42]. Recently, HIF-1α is considered as an inflammation switch which could positive regulated by mTOR [36]. Moreover, it could promote the expression of VEGF, which promotes bronchoalveolar vascular permeability and leads to pulmonary edema in ALI [43]. Furthermore, inhibition of HIF-1α reduces inflammation-related tissue damage in ALI [44, 45]. Thus, inhibition of PI3K/AKT/mTOR/HIF-1α/VEGF signaling pathway could reduce the injuries of ALI such as exaggerated inflammatory response and pulmonary edema.

It should be noted that although many pathways and GO terms from the network pharmacology approach were also present in the RNA-seq analysis by comparing the results of the KEGG and GO enrichment analysis, the top GO terms profiles seem not consistent in our studies. This might be due to the differences between the two approaches such as data origination, gene numbers, model algorithm, and etc., which would affect GO terms permutations or ranks [46].

In general, the network pharmacology has benefits for analyzing network targets and multi-components as a guided theoretical science, but it’s unable to distinguish inhibitory effects from activation effects [47]. RNA-seq could compensate for these shortcomings. However, with acquisition of huge numbers of DEGs it is confusing to obtain exact or reliable targets, especially in limited sample size [48]. In the work, through combination of the two approaches, we conveniently focused on 57 key targets. Most of these targets were possibly not the hot points analyzed by individual approach. However, while conducting a GSEA analysis enriched 57 targets (constructed as a gene set database) into an ALI related gene dataset from GEO database (as an expression dataset), these key targets as a whole set were of significantly biological relevance. Therefore, the combined strategy would be benefit to screening out some less conspicuous targets which might be neglected in single approach analysis. Nevertheless, there still existed some limitations in the work, such as limited number of RNA-seq samples per group, use of genetic databases to predict XCD targets, single time-point for RNA-seq analysis (may miss target genes but should hit target) [49].

## Conclusions

In conclusion, the protective mechanism of XCD on ALI treatment was probably through a crucial mechanism involved in downregulation of PI3K/mTOR/HIF-1α/VEGF signaling pathway.

## Supplementary Information

**Additional file 1.** **Table S1.** The primer sequences for qRT-PCR analysis. **Table S2.** The physicochemical and drug-like properties. **Table S3.** Candidate compounds and potential targets in XCD. **Table S4.** The ALI related targets. **Table S5.** Network Pharmacology analysis of XCD. **Table S6.** Differently expressed genes were identified between the Model and XCD groups in RNA-Seq. **Table S7.** The Gene and FPKM values of DEGs between Model and XCD group with significantly expression. **Table S8.** Analysis results of 57 target genes' PPI Network. **Table S9.** List of the KEGG pathway of kernel genes. **Table S10.** GSEA details of 57 target genes enrichment in the dataset of human lung microvascular endothelial cells (HMVEC) stimulated with or without LPS for 8 hours.

## Data Availability

The datasets generated during and/or analyzed during the current study are available from the corresponding author on reasonable request.

## Abbreviations

ALI: Acute lung injury
ANOVA: A one-way analysis of variance
ARDS: Acute respiratory distress syndrome
BALF: Bronchoalveolar lavage fluid
DAVID: Database for annotation, visualization, and integrated discovery
De: Degree
DEGs: Differentially expressed genes
DL: Drug-likeness
FDR: False discovery rate
FPKM: Fragments per kilobase million
GO: Gene ontology
GSEA: Gene set enrichment analysis
HIF-1α: Hypoxia-inducible factor-1α
HMVEC: Human lung microvascular endothelial cells
KEGG: Kyoto encyclopedia of genes and genomes
IL: Interleukin
LPS: Lipopolysaccharide
LSD: The least-significant difference
mTOR: Mammalian target of rapamycin
OB: Oral bioavailability
PI3K: Phosphoinositide 3-kinase
PPI: Protein–protein interaction
PTEN: Phosphate and tension homology deleted on chromosome ten
RNA-seq: RNA sequencing
TC: Topological coefficient
TCM: Traditional Chinese medicine
TCMIP: Integrative pharmacology-based research platform of TCM
TCMSP: TCM systems pharmacology database
TNF: Tumor necrosis factor
VEGF: Vascular epidermal growth factor
XCD: Xuan-bai-cheng-qi decoction

## References

[1] Matthay MA, Zemans RL, Zimmerman GA, Arabi YM, Beitler JR, Mercat A (2019). Acute respiratory distress syndrome. Nat Rev Dis Primers.

[2] Butt Y, Kurdowska A, Allen TC (2016). Acute lung injury: a clinical and molecular review. Arch Pathol Lab Med.

[3] Fan E, Brodie D, Slutsky AS (2018). Acute respiratory distress syndrome: advances in diagnosis and treatment. JAMA.

[4] Patel VJ, Biswas RS, Mehta HJ, Joo M, Sadikot RT (2018). Alternative and natural therapies for acute lung injury and acute respiratory distress syndrome. Biomed Res Int.

[5] Jin J, Zhang H, Li D, Jing Y, Sun Z, Feng J (2019). Effectiveness of Xin Jia Xuan Bai Cheng Qi Decoction in treating acute exacerbation of chronic obstructive pulmonary disease: study protocol for a multicentre, randomised, controlled trial. BMJ Open.

[6] Liu M, Zhong X, Li Y, Zheng F, Wu R, Sun Y (2014). Xuan Bai Cheng Qi formula as an adjuvant treatment of acute exacerbation of chronic obstructive pulmonary disease of the syndrome type phlegm-heat obstructing the lungs: a multicenter, randomized, double-blind, placebo-controlled clinical trial. BMC Complement Altern Med.

[7] Chinese Pharmacopoeia Commission (2015). Pharmacopoeia of the people’s republic of China (2015).

[8] Meng FS. Clinical effects of modified Xuanbai chengqi decoction on the treatment of severe pneumonia with phlegm heat obstructing lung type. Hebei J Tradit Chin Med. 2016; 38:92–4. https://kns.cnki.net/kcms/detail/detail.aspx?dbcode=CJFD&dbname=CJFDLAST2016&filename=HBZY201601032&v=Q43StBfZXM7YM04VAqJfS2IDkvZ48uAJpPoEsILI1y0IVUuf5ZqBf%25mmd2BGtT6urZ4FV.

[9] Mao Z, Wang H (2016). Effects of Xuanbai Chengqi decoction on lung compliance for patients with exogenous pulmonary acute respiratory distress syndrome. Drug Des Devel Ther.

[10] China National Health Commission. Influenza ttraining manual for medical staff. 2019. http://www.nhc.gov.cn/yzygj/s7653p/201911/a577415af4e5449cb30ecc6511e369c7/files/2863910c9db748c18408fd68e55911ea.pdf.

[11] China National Health Commission. Diagnosis and treatment protocol for COVID-19 (Trial Version 7). 2020. http://www.nhc.gov.cn/yzygj/s7653p/202002/3b09b894ac9b4204a79db5b8912d4440/files/7260301a393845fc87fcf6dd52965ecb.pdf.

[12] Zhang HY, Wang LX, Yang AD, SU ZH, Wu ZH, et al. Effects of xuanbai chengqi decoction on lung of expressions of MD-2 and MyD88 mRNA and protein in rats with acute lung injury caused by lipopolysaccharide. J Liaoning Univ Tradit Chin Med. 2013;15:36–9. https://kns.cnki.net/kcms/detail/detail.aspx?dbcode=CJFD&dbname=CJFD2013&filename=LZXB201309014&v=k8SazDPOG6ssWVhxiM4vvjrc0qmNWCGJCL8pZgem7z%25mmd2BwhJQIBRkAqll%25mmd2Fvb79%25mmd2BVNF.

[13] Su ZH, Yang AD, Wang LX, Guo YJ, Wu ZH. Effect of xuanbai chengqi decoction on CD14 and NF-κB mRNA expressions in rat LPS-induced acute lung injury model. Chin J Exp Tradit Med Form. 2012; 18:121–5. http://www.syfjxzz.com/zgsyfjxzz/article/abstract/20120538?st=article_issue.

[14] Su ZH, Yang AD, Wang LX, Guo YJ, Wu ZH. The effect of xuanbai chengqi decoction on LBP and TLR4 mRNA expressions in acute lung injured rat model. J Nanjing Univ Tradit Chin Med. 2013; 29:155–8. http://xb.njucm.edu.cn/jnutcmns/ch/reader/view_abstract.aspx?file_no=ZR20130216&flag=1.

[15] Liu ZH, Sun XB (2012). Network pharmacology: new opportunity for the modernization of traditional Chinese medicine. Yao Xue Xue Bao.

[16] Jiang Y, Zhu Y, Zhen T, Li J, Xing K, He L (2020). Transcriptomic analysis of the mechanisms of alleviating renal interstitial fibrosis using the traditional Chinese medicine Kangxianling in a rat model. Sci Rep.

[17] Finotello F, Di Camillo B (2015). Measuring differential gene expression with RNA-seq: challenges and strategies for data analysis. Brief Funct Genomics.

[18] Luo TT, Lu Y, Yan SK, Xiao X, Rong XL, Guo J (2020). Network Pharmacology in research of Chinese medicine formula: methodology, application and prospective. Chin J Integr Med.

[19] Cai F, Bian Y, Wu R, Sun Y, Chen X, Yang M, Zhang Q (2019). Yinchenhao decoction suppresses rat liver fibrosis involved in an apoptosis regulation mechanism based on network pharmacology and transcriptomic analysis. Biomed Pharmacother.

[20] Ru J, Li P, Wang J, Zhou W, Li B, Huang C, Li P (2014). TCMSP: a database of systems pharmacology for drug discovery from herbal medicines. J Cheminform.

[21] Xu HY, Zhang YQ, Liu ZM, Chen T, Lv CY, Tang SH (2019). ETCM: an encyclopaedia of traditional Chinese medicine. Nucleic Acids Res.

[22] Wang N, Zheng Y, Gu J, Cai Y, Wang S, Zhang F, Chen J (2017). Network-pharmacology-based validation of TAMS/CXCL-1 as key mediator of XIAOPI formula preventing breast cancer development and metastasis. Sci Rep.

[23] Safran M, Dalah I, Alexander J, Rosen N, Iny Stein T, Shmoish M (2010). GeneCards version 3: the human gene integrator. Database (Oxford)..

[24] UniProt C (2019). UniProt: a worldwide hub of protein knowledge. Nucleic Acids Res.

[25] Amberger JS, Bocchini CA, Schiettecatte F, Scott AF, Hamosh A (2015). OMIM.org: online mendelian inheritance in man (OMIM(R)), an online catalog of human genes and genetic disorders. Nucleic Acids Res.

[26] Bauer-Mehren A, Rautschka M, Sanz F, Furlong LI (2010). DisGeNET: a Cytoscape plugin to visualize, integrate, search and analyze gene-disease networks. Bioinformatics.

[27] Huang DW, Sherman BT, Lempicki RA (2009). Bioinformatics enrichment tools: paths toward the comprehensive functional analysis of large gene lists. Nucleic Acids Res.

[28] Kanehisa M, Furumichi M, Tanabe M, Sato Y, Morishima K (2017). KEGG: new perspectives on genomes, pathways, diseases and drugs. Nucleic Acids Res.

[29] Sun L, Dong S, Ge Y, Fonseca JP, Robinson ZT, Mysore KS (2019). DiVenn: an interactive and integrated web-based visualization tool for comparing gene lists. Front Genet.

[30] Von MC, Jensen LJ, Snel B, Hooper SD, Krupp M, Foglierini M (2005). STRING: known and predicted protein-protein associations, integrated and transferred across organisms. Nucleic Acids Res.

[31] Subramanian A, Kuehn H, Gould J, Tamayo P, Mesirov JP (2007). GSEA-P: a desktop application for gene set enrichment analysis. Bioinformatics.

[32] Pandey MK, Sung B, Ahn KS, Kunnumakkara AB, Chaturvedi MM, Aggarwal BB (2007). Gambogic acid, a novel ligand for transferrin receptor, potentiates TNF-induced apoptosis through modulation of the nuclear factor-kappaB signaling pathway. Blood.

[33] Tuo CD, Yuan BH, Liu Y, Guo SM. Effects of XuanBai ChengQi Decoction enema on ET and I-FABP of the patients with acute lung injury. West J Tradit Chin Med. 2017; 30:110–1. https://kns.cnki.net/kcms/detail/detail.aspx?dbcode=CJFD&dbname=CJFDLAST2017&filename=GSZY201711034&v=FqGxCHRirhjTe7RQN5tHjyPPDPIOqmFrpfzlldSAesBbo3vFQqkCHRSQULTGUbIF.

[34] Wu Z, Tan B, Zhang H, Guo Y, Tu Y, Qiu F (2017). Effects of sodium houttuyfonate on pulmonary inflammation in COPD model rats. Inflammation.

[35] Zhu M, Hong D, Bao Y, Wang C, Pan W (2013). Oridonin induces the apoptosis of metastatic hepatocellular carcinoma cells via a mitochondrial pathway. Oncol Lett.

[36] Li X, Shan C, Wu Z, Yu H, Yang A, Tan B (2020). Emodin alleviated pulmonary inflammation in rats with LPS-induced acute lung injury through inhibiting the mTOR/HIF-1alpha/VEGF signaling pathway. Inflamm Res.

[37] Zhang Q, Qian YM. Clinical observation of Xuan Bai Cheng Qi decoction on senile community acquired pneumonia with phlegm heat obstructing lung type. Hebei J Tradit Chin Med. 2018; 40:1316–20. https://kns.cnki.net/kcms/detail/detail.aspx?dbcode=CJFD&dbname=CJFDLAST2018&filename=HBZY201809008&v=ETDkXDUuVkw2XaFFfFo%25mmd2B1i00eCCNFLQ2WJsxSWz3K3%25mmd2F15sBipEHkybgSVna5tLHM.

[38] Mu S, Zhang J, Du S, Zhu M, Wei W, Xiang J (2021). Gut microbiota modulation and anti-inflammatory properties of Xuanbai Chengqi decoction in septic rats. J Ethnopharmacol.

[39] Liu C, Yin Z, Feng T, Zhang M, Zhou Z, Zhou Y (2021). An integrated network pharmacology and RNA-Seq approach for exploring the preventive effect of Lonicerae japonicae flos on LPS-induced acute lung injury. J Ethnopharmacol.

[40] Zhao H, Ma Y, Zhang L (2018). Low-molecular-mass hyaluronan induces pulmonary inflammation by up-regulation of Mcl-1 to inhibit neutrophil apoptosis via PI3K/Akt1 pathway. Immunology.

[41] Hsieh YH, Deng JS, Chang YS, Huang GJ (2018). Ginsenoside Rh2 ameliorates lipopolysaccharide-induced acute lung injury by regulating the TLR4/PI3K/Akt/mTOR, Raf-1/MEK/ERK, and Keap1/Nrf2/HO-1 signaling pathways in mice. Nutrients.

[42] Wang SH, Li LH, Zou DM, Zheng XM, Deng J (2020). Roles of the mammalian target of rapamycin (mTOR) signaling pathway in the repair of hyperoxia-induced acute lung injury. Adv Clin Exp Med.

[43] McClendon J, Jansing NL, Redente EF, Gandjeva A, Ito Y, Colgan SP (2017). Hypoxia-inducible factor 1alpha signaling promotes repair of the alveolar epithelium after acute lung injury. Am J Pathol.

[44] Lin F, Pan LH, Ruan L, Qian W, Liang R, Ge WY (2015). Differential expression of HIF-1alpha, AQP-1, and VEGF under acute hypoxic conditions in the non-ventilated lung of a one-lung ventilation rat model. Life Sci.

[45] Zhang X, Li J, Li C, Li Y, Zhu W, Zhou H (2015). HSPA12B attenuates acute lung injury during endotoxemia in mice. Int Immunopharmacol.

[46] Weiler S, Ademokun JA, Norton JD (2015). ID helix-loop-helix proteins as determinants of cell survival in B-cell chronic lymphocytic leukemia cells in vitro. Mol Cancer.

[47] Wu R, Dong S, Cai FF, Chen XL, Yang MD, Liu P, et al. Active compounds derived from fuzheng huayu formula protect hepatic parenchymal cells from apoptosis based on network pharmacology and transcriptomic analysis. Molecules. 2019; 24. 10.3390/molecules24020338 PMC6358846 30669350

[48] Wang M, Bu X, Luan G, Lin L, Wang Y, Jin J (2020). Distinct type 2-high inflammation associated molecular signatures of chronic rhinosinusitis with nasal polyps with comorbid asthma. Clin Transl Allergy.

[49] Shankar P, Geier MC, Truong L, McClure RS, Pande P, Waters KM, et al. Coupling genome-wide transcriptomics and developmental toxicity profiles in zebrafish to characterize polycyclic aromatic hydrocarbon (PAH) hazard. Int J Mol Sci. 2019; 20. 10.3390/ijms20102570 PMC6566387 31130617

